# Chromosome-level genome assembly of golden pompano (*Trachinotus ovatus*) in the family Carangidae

**DOI:** 10.1038/s41597-019-0238-8

**Published:** 2019-10-22

**Authors:** Dian-Chang Zhang, Liang Guo, Hua-Yang Guo, Ke-Cheng Zhu, Shang-Qi Li, Yan Zhang, Nan Zhang, Bao-Suo Liu, Shi-Gui Jiang, Jiong-Tang Li

**Affiliations:** 1 Key Laboratory of South China Sea Fishery Resources Exploitation & Utilization, Ministry of Agriculture and Rural Affairs, South China Sea Fisheries Research Institute, Chinese Academy of Fishery Sciences, 231 Xingang Road West, Haizhu District, Guangzhou 510300 China; 2 Key Laboratory of Aquatic Genomics, Ministry of Agriculture and Rural Affairs, CAFS Key Laboratory of Aquatic Genomics and Beijing Key Laboratory of Fishery Biotechnology, Chinese Academy of Fishery Sciences, Beijing, 100141 China

**Keywords:** DNA sequencing, Transcriptomics

## Abstract

Golden pompano (*Trachinotus ovatus*), a marine fish in the Carangidae family, has a wide geographical distribution and adapts to severe environmental rigours. It is also an economically valuable aquaculture fish. To understand the genetic mechanism of adaption to environmental rigours and improve the production in aquaculture, we assembled its genome. By combination of Illumina and Pacbio reads, the obtained genome sequence is 647.5 Mb with the contig N50 of 1.80 Mb and the scaffold N50 of 5.05 Mb. The assembly covers 98.9% of the estimated genome size (655 Mb). Based on Hi-C data, 99.4% of the assembled bases are anchored into 24 pseudo-chromosomes. The annotation includes 21,915 protein-coding genes, in which 95.7% of 2,586 BUSCO vertebrate conserved genes are complete. This genome is expected to contribute to the comparative analysis of the Carangidae family.

## Abstract (summary)

**Table:** 

Measurement(s)	DNA • chromosome conformation capture assay • transcription profiling assay
Technology Type(s)	DNA sequencing • Hi-C • RNA sequencing
Factor Type(s)	organism part
Sample Characteristic - Organism	Trachinotus ovatus
Sample Characteristic - Environment	ocean biome

Machine-accessible metadata file describing the reported data: 10.6084/m9.figshare.9933515

## Background & Summary

The golden pompano, *Trachinotus ovatus* (Linnaeus 1758), belongs to Carangiformes and is widely distributed in tropical and subtropical oceans^1^. From a biogeographic perspective, this fish readily tolerates different environments. In addition, this fish has been one of the most importantly economic marine fish in China^2^. However, overfishing, diseases, and degeneration of genetic diversity have caused serious economic losses in *T*. *ovatus* production^3^. Many solutions, including selective breeding^4^, identification of trait-associated genes^5^, and dietary supplementation^6^, are adopted to overcome these problems and improve the production.

The golden pompano is a marine fish in the Carangidae family. One characteristic of this family is the indistinguishable sex chromosomes^7^. It is speculated that sex chromosomes in this family has not been largely differentiated, distinct from those with well-differentiated sex chromosomes^8^. Therefore, fish in this family could be used to analyse the initial evolution status of the sex-determination system. Another characteristic of this family is tolerance to high turbidity, rapid pH changes and low dissolved oxygen concentrations and crowding^9^. The Carangidae fish are potential candidates to study resistance to stress.

**Fig. 1:**
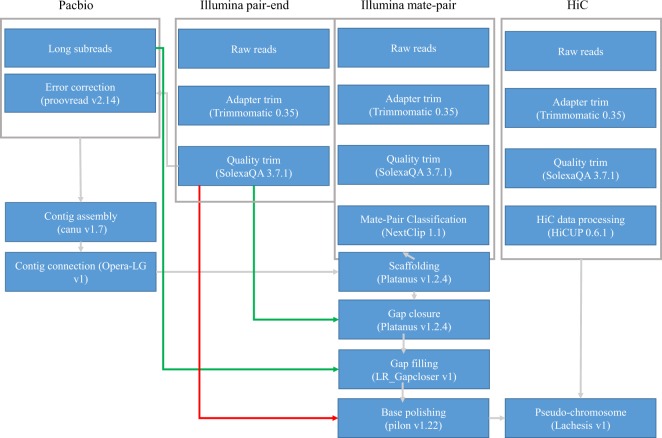
The pipelines of the chromosome-level pompano genome assembly.

**Fig. 2:**
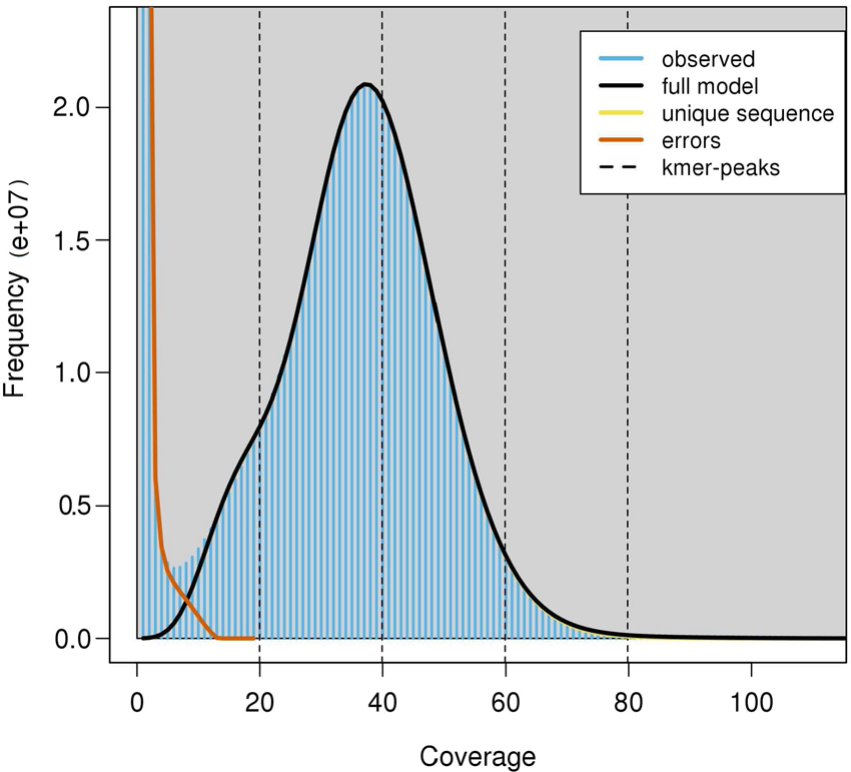
The K-mer distribution of Illumina paired-end reads using GenomeScope based on k value of 31. Frequency distribution of k-mers of different occurrences in two pair-end libraries. K-mer occurrences (x axis) were plotted against their frequencies (y axis).

A high-quality genome assembly is necessary to understand the functional, ecological and evolutional genomics of this species and other fish in the Carangidae family. In the present study, we presented a chromosome-level genome assembly of pompano using Illumina sequencing, Pacbio sequencing, and Hi-C technology (Fig. 1). We produced 105 Gb of cleaned Illumina reads of genomic DNA, 16.9 Gb Pacbio long reads, and 114.8 Gb cleaned data from a Hi-C library. The genome size was estimated to be 655 Mb (Fig. 2). A 647.5 Mb assembly of pompano was generated. The contig N50 length and scaffold N50 length were 1.80 Mb and 5.05 Mb, respectively. Based on 114.8 Gb Hi-C data, 99.4% of the assembly were anchored into 24 pseudo-chromosomes. The annotation includes 21,915 protein-coding genes.

The coverage of the estimated genome size (98.9%), the proportion of bases anchored to the pseudo-chromosomes (99.4%), the RNA-seq alignment ratio (90.5%), the proportion of pompano protein-coding genes having homologues (97.5%), and the ratio of complete BUSCO vertebrate genes (95.7%) all indicate that this genome assembly is of high quality. The genome assembly and its annotated information would be useful for studies on environmental adaptions, resistance to disease and sex determination. This genome has already proven to be useful to mine functional genes underlying resistance to disease^10,11^. It is the first chromosome-level genome in the Carangidae family and is expected to contribute to the study of the diversity, speciation, and evolution of this family.

## Methods

### Ethics statement

The sampled fish in this study was permitted by the Animal Care and Use Committee of South China Sea fisheries Research Institute, Chinese Academy of fishery Sciences (No. SCSFRI96-253) and performed by the regulations and guidelines established with this committee.

### Sampling and sequencing

A female pompano was collected in Xincun Bay, Hainan, China. Total genomic DNA was extracted using a DNA Extraction Kit (MAGEN Company, Guangdong, China) following the manufacturer’s protocols. The quality and quantity of total DNA were determined by a NanoDrop 2000 spectrophotometer (Thermo Fisher Scientific, Wilmington, DE, USA). We constructed two paired-end libraries (insert sizes of 500 and 700 bp) and three mate-pair libraries (insert sizes of 3, 5, and 14 kb) according to Illumina standard procedures (Illumina, San Diego, CA, USA). The libraries were sequenced on a HiSeq 2500 system with 250 bp PE mode or 100 bp PE mode (Table 1).

**Table 1:** Data statistics of whole genome sequencing reads of pompano.

Platform	Insert size	Clean pairs	Total bases	Genome coverage (X)	SRA accession
Illumina	500 bp	44,554,312	19,894,674,143	30.3	SRR8185380
	700 bp	94,147,131	15,691,188,500	23.9	SRR8185379
	3 K bp	24,639,173	5,597,129,699	8.5	SRR8185378
	5 K bp	22,753,897	5,688,834,998	8.6	SRR8185382
	14 K bp	149,292,822	28,171,641,480	42.9	SRR8185385
Hi-C (Illumina X ten)		382,798,592	114,839,577,600	175.1	SRR8168440
Pacbio		2,278,176	16,879,861,540	25.7	SRR7943174
	Total	272,622,581	206,762,907,960	315.3	

The extracted DNA molecules were also used to construct two 20 kb libraries following the PacBio manufacturing protocols (Pacific Biosciences, CA, USA). The libraries were then sequenced with two cells on PacBio Sequel platform (Table 1).

The Hi-C technique has been applied into constructing chromosome-level assemblies^12,13^. We prepared a Hi-C library for the chromosome assembly of pompano following the strategy of Rao *et al*.^14^. Briefly, the blood sample was fixed with fresh formaldehyde and then DNA-protein bonds were created. The restriction enzyme of *Mbo* I digested the DNA and the overhanging 5′ ends of the DNA fragments were repaired with a biotinylated residue. The fragments close to each other in the nucleus during fixation were ligated. The Hi-C fragments were further sheared by sonication into smaller fragments of ~350 bp in size, which were then pulled-down with streptavidin beads. The Hi-C library for Illumina sequencing was prepared according to the manufacturer’s standard procedures. The library was sequenced on the Illumina HiSeq X Ten platform with 150 bp PE mode.

Eight tissues (blood, liver, muscle, brain, spleen, fin, ovary and stomach) were collected. Total RNA from each tissue was extracted and treated with DNase I (Thermo Fisher Scientific, Wilmington, DE, USA) to remove genomic DNA. The RNA integrity of each tissue was confirmed with a Bioanalyzer 2100 (Agilent Technologies, Santa Clara, USA). For each tissue, we constructed two RNA-sequencing libraries with an insert size of 300 bp and then sequenced them on the Illumina HiSeq platform with 150 bp PE mode.

### Read filtration and genome size estimation

The genomic sequencing reads from five Illumina libraries were first cleaned to remove the adapters using Trimmomatic-0.35^15^. Then the quality trimming was performed using SolexaQA v3.7.1^16^ to filter the low-quality bases and short reads <25 bp. We produced 105 Gb of cleaned Illumina reads of genomic DNA. Especially, the reads from the mate-pair libraries, were further subjected to classification of the mate pairs using the Nextclip v1.1^17^.

Pacbio sequencing generated ~16.9 Gb long reads (Table 1). The mean and N50 length were 7.4 and 12.2 kb, respectively. We corrected the Pacbio long reads with reads from two Illumina paired-end libraries using proovread v2.14^18^. Additionally, the paired-end reads of the Hi-C library were trimmed by filtering adapters and removing reads of low quality with Trimmomatic-0.35^15^ and SolexaQA v3.7.1^16^, respectively. 382 million cleaned reads with the total bases of 114.8 Gb were generated from the Hi-C library.

Before genome assembly and gene annotation, we estimated the genome size by the k-mer analysis using 35.58 Gb filtered reads from the two paired-end Illumina libraries (500 bp and 700 bp libraries). The number of effective k-mers and the peak depth of a series of k values (17, 19, 21, 23, 25, 27, 29, and 31) were produced using Jeffyfish (v2.2)^19^ with the C-setting. The genome size was estimated following the formula Genome_Size = (Total k-mers - Erroneous k-mers)/Peak^20^. The maximal genome size was calculated to be 655 Mb when a k-mer size was 31 (Table 2). The estimated genome size was within the range of previously reported sizes of other Carangidae fish (614.2 Mb~716.4 Mb, Table 3). Hence, the sequencing coverages of the cleaned Illumina reads, Pacbio reads, and Hi-C data were 114.5, 25.7, and 175.1-fold, respectively. The rate of genome heterozygosity estimated by GenomeScope (v1.0.0)^21^ was around 0.31% (Fig. 2). The low heterozygosity indicated this genome to be homozygous.

**Table 2:** Estimation of genome size of pompano by k-mer analysis.

K	Total number of k-mers	Number of erroneous k-mers	Peak in Jellyfish counting	Estimated genome size (Mb)
17	30,359,515,882	1,700,273,328	45	636.9
19	29,905,858,631	2,266,172,955	43	642.8
21	29,425,980,179	2,419,537,116	42	643.0
23	28,931,567,876	2,494,020,191	41	644.8
25	28,427,735,494	2,544,415,369	40	647.1
27	27,917,344,738	2,581,038,454	39	649.6
29	27,402,087,718	2,606,597,782	38	652.5
31	26,882,868,388	2,621,598,458	37	655.7

**Table 3:** Comparisons of other published Carangiformes assemblies.

Order	Carangiformes						
Family	Carangidae						Echeneidae
Species	*Trachinotus ovatus*	*Seriola quinqueradiata* ^57^	*Seriola dumerili* ^58^	*Seriola lalandi* dorsalis^59^	*Seriola rivoliana* ^60^	*Seriola lalandi* ^61^	*Echeneis naucrates* ^62^
Assembled Size (Mb)	647.5	639.2	672.1	716.4	661.8	614.2	544.2
Scaffold N50 size (Mb)	5.05	5.61	5.81	1.27	9.51	0.411	NA
Total scaffolds	373	384	34,656	99,598	1,343	7,606	NA
Pseudo-chromosome number	24	NA	NA	NA	NA	NA	24
Average pseudo-chromosome length (Mb)	26.8	NA	NA	NA	NA	NA	22.5
Number protein-coding genes	21,915	NA	22,083	25,802	NA	NA	21,288
Average CDS length	1,608	NA	1,806	1,647	NA	NA	1,863
Average exon number	10.4	NA	11.0	9.96	NA	NA	11.2
Average exon length	275	NA	248	271	NA	NA	267

### Hybrid assembly, scaffolding, and chromosome anchoring

The error-corrected long reads were assembled using Canu v1.7^22^ with the default parameters of correctedErrorRate as 0.039. The contigs were further connected into longer contigs with the error-corrected long reads using Opera-LG^23^. The contigs were further scaffolded using mate-pair libraries, and the gaps in the scaffolds were closed with reads from the paired-end libraries using Platanus v1.2.4^24^. The gaps in the assemblies were further filled with the raw long reads using LR_Gapcloser v1.0^25^. The final genome sequences were polished by pilon v1.22^26^ using cleaned Illumina short reads to correct errors in base level. A ~647.5 Mb genome assembly of pompano with 373 scaffolds was constructed. The assembly covered 98.9% of estimated genome regions. The contig N50 length and scaffold N50 length were 1.80 Mb and 5.05 Mb, respectively. A total of 137 scaffolds, longer than 1.26 Mb, covered over 90% of the assembly (Table 2).

**Fig. 3:**
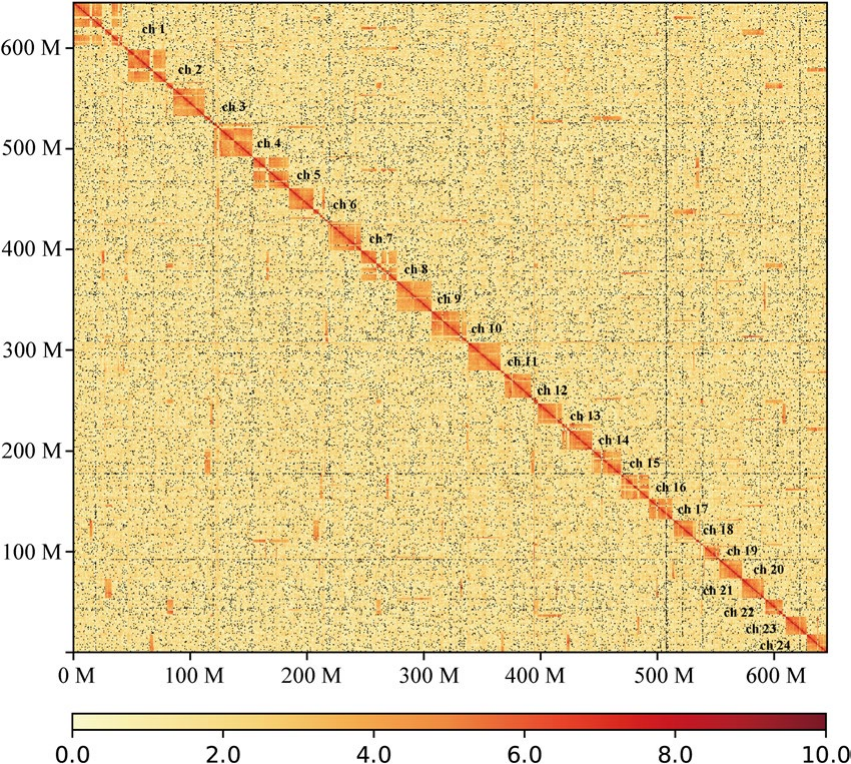
Hi-C chromosome contact map. Each block represents a Hi-C contact between two genomic loci within a 100-kb window. Darker color of a block indicates higher contact intensity.

To anchor scaffolds into pseudo-chromosomes, HiCUP v0.6.1^27^ was firstly used to map and process the reads from the Hi-C library. Two reads of pairs were mapped to the polished scaffolds using Bowtie 2^28^ with the default parameters. If both two reads from one pair were uniquely mapped to the assembly, this pair was retained for the downstream filtration. HiCUP removed invalid pairs which were generated from contiguous sequences, circularization, dangling ends, internal fragments, re-ligation, PCR duplication, and fragments of wrong size. Based on the refined alignments, we clustered 321 scaffolds into pseudo-chromosomes using Lachesis v1.0^29^. It is reported that pompano genome consists of 24 chromosomes by linkage group analysis^30^ and karyotyping^31^. Therefore, the pseudo-chromosome number was set as 24. Finally, Lachesis ordered and oriented 259 scaffolds into 24 pseudo-chromosomes, corresponding to 69.4% and 99.4% of the assembly by sequence number and base count, respectively. The average pseudo-chromosome length was 26.84 Mb. The unanchored 114 scaffolds were much short with an average length of 33.3 kb, covering only 0.6% of the assembly. To validate the correction of the Hi-C scaffolding to pseudo-chromosome level, we constructed an interaction matrix with cleaned reads from the Hi-C library using HiC-Pro^32^ (default parameters and LIGATION_SITE = GATC). The genome was divided into bins of equal size of 100 Kb, and the number of contacts was determined between each pair of reported bins. A contact map plotted with HiCPlotter^33^ confirmed the genome structure and quality (Fig. 3). Compared with other Carangidae fish, it is the first chromosome-level assembly in this family (Table 3).

### *De novo* repeat prediction and classification

Before predicting protein-coding genes, we masked the repetitive regions of the assembly using a combination of *ab initio* and homology-based approaches. RepeatModeler v1.0.11 (http://www.repeatmasker.org/RepeatModeler/) was used to construct a pompano-specific repeat library consisting of 1,134 consensus repeats. By using RepeatMasker v4.0.7 (http://repeatmasker.org/cgi-bin/WEBRepeatMasker), the repeat regions of this assembly were masked first with the Repbase teleost repeat library^34^ and then with the pompano-specific library. The results from the stepwise method identified 131.22 Mb of repeat sequences, included 109.9 Mb of interspersed repeats and 21.1 Mb of tandem repeats. Among classified interspersed repeats, DNA transposons were more abundant than retrotransposons. The repeats accounted for 20.25% of the assembly (Table 4), close to that of published diploid fish genomes^35–37^.

**Table 4:** Repeat content in pompano genome.

Repeat elements	Copies	Bases	Percent (%)
**Interspersed repeats**			
SINE	11,964	1,473,642	0.22
Penelope	2,054	373,482	0.06
LINE	54,917	13,503,181	2.08
LTR	15,038	2,965,180	0.46
DNA transposon	161,301	22,551,263	3.48
Unclassified	435,045	69,429,000	10.71
Subtotal	680,319	109,922,266	16.96
**Tandem repeats**			
Satellites	1,037	167,798	0.026
Simple repeats	415,200	18,131,460	2.80
Low complexity	50,191	2,814,637	0.43
Subtotal	466,428	21,113,895	3.26
**Small RNA**	2,167	188,301	0.029
**Total**	1,148,914	131,224,462	20.25

### Gene prediction and functional annotation

Based on the repeat-masked assembly, we predicted gene models by integrating *ab initio* predictions, homologue prediction, and RNA-seq models. First, Fgenesh^38^ was used to construct *de novo* gene models. Second, we aligned fish proteins from the Ensembl database^39^ to the assembly using BLAT^40^. All fish proteins annotated in Ensembl database were downloaded to construct an Ensembl fish protein set. The proteins having alignments with over 70% coverage were re-aligned to the assembly using GeneWise^41^ for accurately spliced alignments. Third, a total of 32 Gb of clean RNA-seq reads from eight tissues trimmed by Trimmomatic-0.35^16^ and SolexaQA v3.7.1^16^ were used to construct RNA-seq based gene models. RNA-seq reads were mapped to the genome using HISAT2^42^, and the alignments were input to Cufflinks^43^ to predict transcripts. All three sets of gene models were merged to form a comprehensive consensus gene set using Cuffmerge^43^. For each model, the longest transcript was selected as the representative transcript. The coding region and protein sequence of the representative transcript were predicted using Transdecoder (https://transdecoder.github.io/). A consensus pompano gene set consisted of 21,915 protein-coding genes. The protein-coding gene number and structures were comparable with that of published Carangiformes genomes (Table 3).

Then we searched for homologues of pompano proteins by aligning them against the Swiss-Prot database, TrEMBL database^44^ and Ensembl fish protein set with Blastp (e value of 10^−5^). Homologue searches found that 21,365 of pompano genes had homologues in at least one database (Table 5). The KEGG biological pathways and Gene Ontology terms of each gene were annotated using the KEGG Automatic Annotation Server^45^ and Blast2GO^46^, respectively. Among the identified protein-coding genes, 20,594 genes were annotated to have at least one Gene Ontology (GO) term, and 7,956 genes were mapped to KEGG pathways. Finally, 21,365 genes (97.5%) were assigned to at least one of five databases (Table 5).

**Table 5:** Annotation of pompano genes to different databases.

Type	Database	Assigned gene number
Homolog	Ensembl	21,277
	SwissProt	19,794
	TrEMBL	21,356
	Total	21,365
Gene Ontology		20,594
KEGG pathway		7,956
Total		21,365

### Quality assessment of genome assembly and gene annotation

**Fig. 4:**
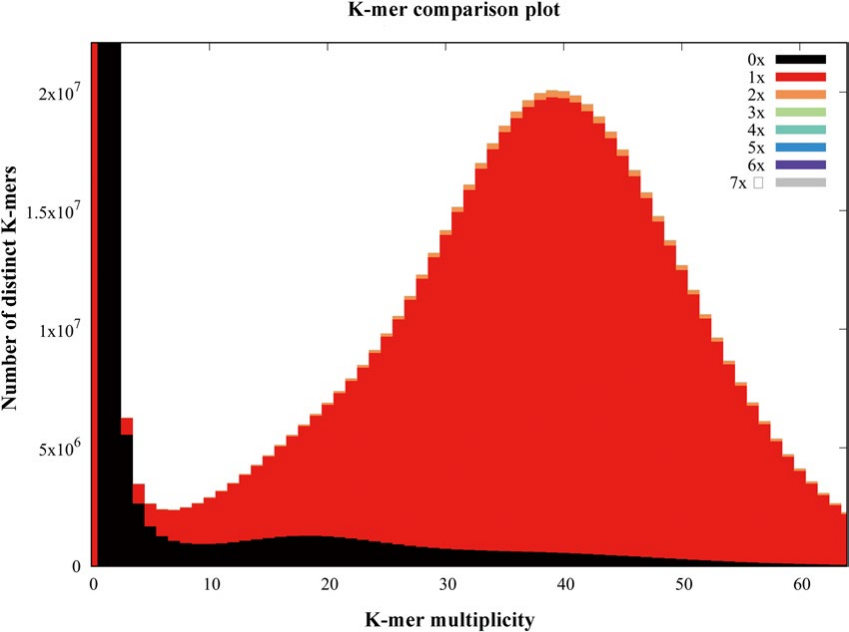
K-mer spectra copy number plot. Different color on the stacked bars represents copy number on the assembly. Frequency counts (spectral distribution) are computed on the Illumina paired-end reads.

**Fig. 5:**
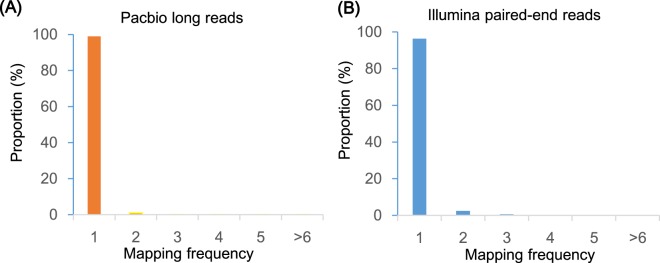
Alignment frequency distribution of Pacbio long reads and Illumina short reads.

**Fig. 6:**
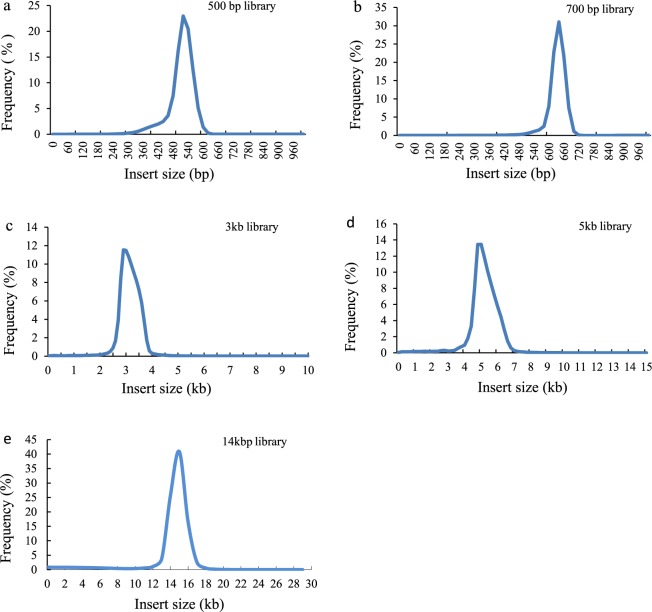
Distribution of insert sizes of sequencing reads in five libraries.

The quality of the assembly was evaluated using multiple indicators. (1) To estimate the quality value (QV) of the assembly, the cleaned reads from two paired-end libraries were mapped to the assembly with BWA^47^ and then the pipeup file produced by SAMtools^48^ were input to Referee^49^ to calculate a quality score for every position. Referee provided a higher scoring base to an erroneous position and this reference base was considered to be an error. We estimated that this genome had one error per 1000 base pairs with a quality value of 30. (2) We validated the assembly by comparing the cleaned read spectrum from two paired-end libraries with the copy number in the assembly using KAT toolkit^50^. The k-mer showed the homozygous distribution without a heterozygous peak (Fig. 4), consistent with the low heterozygosity observed by GenomeScope (Fig. 2). The main content occurred once, suggesting that the homozygous regions were not expanded. Furthermore, the absent k-mers (black) at the frequency of average sampling depth was low (Fig. 4), suggesting a high level of assembly completeness. The assembly correctly represented kmer spectrum from the cleaned Illumina reads. (3) We aligned Pacbio long reads to the repeat-masked assembly using Minimap2^51^ and retained those alignments having read coverages over 90%. Almost 98.9% of long reads were uniquely aligned, suggesting that few homozygous contents were duplicated (Fig. 5). The cleaned Illumina reads were aligned to the repeat-masked assembly using BWA^47^. With the coverage threshold of 90%, over 96.3% of reads were uniquely aligned, also supporting few duplicated homozygous contents (Fig. 5). These two distributions were consistent with the main unique content in the KAT analysis. (4) The insert size distributions of paired-end/mate-pair libraries by aligning reads to the genome using BWA^47^ were consistent with the estimated insert sizes (Fig. 6). (5) The clean RNA-seq reads from multiple tissues had an average alignment ratio of 90.5% to the assembly using HISAT2^42^ (Table 6). All the indicators suggested a high-quality genomic resource for the further analysis. The indistinguishable sex chromosome is one characteristic of this family. This chromosome-level assembly would provide a reference to identify sex chromosome and study the evolution of sex chromosome.

**Table 6:** Mapping ratio of RNA-seq reads from eight tissues.

Tissue	Cleaned pairs	Total bases	Alignment ratio	SRA accession
Blood	10,639,911	2,631,736,943	90.67%	SRR8656488
Liver	16,235,470	4,029,392,277	89.20%	SRR8656489
Muscle	14,800,607	3,677,971,940	94.05%	SRR8656490
Brain	14,983,402	3,714,276,260	82.65%	SRR8656491
Spleen	8,778,246	2,178,602,070	93.22%	SRR8656484
Fin	25,750,965	6,390,342,718	93.52%	SRR8656485
Ovary	19,151,732	4,749,798,341	91.98%	SRR8656486
Stomach	18,574,229	4,604,137,153	87.94%	SRR8656487
Total	128,914,562	31,976,257,702	90.49%	

The completeness of pompano genes was evaluated by using BUSCO software^52^. The pompano genes were compared with the 2,586 BUSCO vertebrate conserved gene set. Comparing pompano genes with the vertebrate gene set revealed that 95.7% of the vertebrate genes were identified as complete. The ‘complete and single-copy BUSCOs’ genes accounted for 94.3% of the total genes, and the ‘complete and duplicated BUSCOs’ genes represented 1.4% (Table 7).

**Table 7:** BUSCO evaluation of the pompano genes compared with the vertebrate gene set.

BUSCO benchmark	Number	Percentage (%)
Complete BUSCOs	2,473	95.7%
Complete and single-copy BUSCOs	2,438	94.3%
Complete and duplicated BUSCOs	35	1.4%
Fragmented BUSCOs	45	1.7%
Missing BUSCOs	68	2.6%
Total BUSCO vertebrate genes	2,586	100%

### Comparison of pompano genome with other Carangiformes genomes

**Fig. 7:**
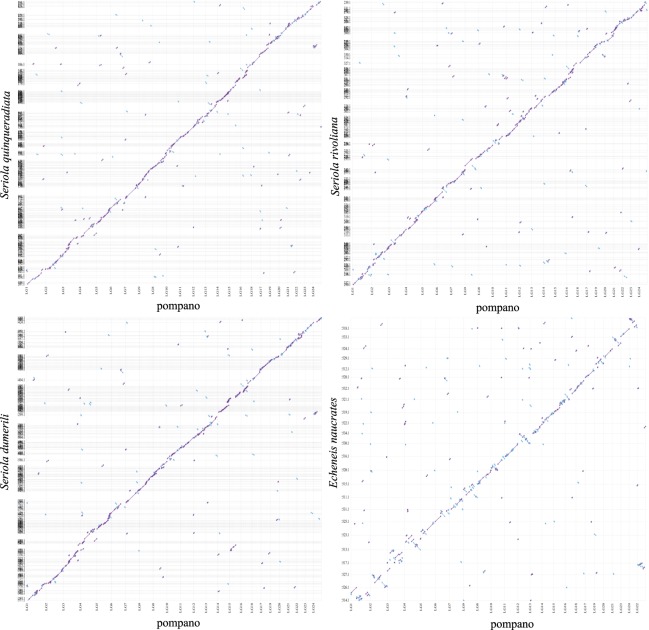
Whole genome plot of four Carangiformes genomes compared to pompano genome. Alignment dot plots show the genome comparisons between four Carangiformes assemblies (*y*-axis) and pompano assembly (*x*-axis). Dotted lines (vertical and horizontal, respectively) are the boundaries of chromosome and of scaffolds in the assemblies. (**a**) Plot between the assemblies of *Seriola quinqueradiata* and pompano. (**b**) Plot between *Seriola rivoliana* assembly and pompano assembly. (**c**) Plot between *Seriola dumerili* assembly and pompano assembly. (**d**) Plot between *Echeneis naucrates* assembly and pompano assembly.

We then compared the pompano genome with other four Carangiformes genomes, including three Carangidae genomes (*Seriola quinqueradiata*, *Seriola dumerili*, and *Seriola rivoliana*) and one Echeneidae genome (*Echeneis naucrates*) using Mashmap2^53^ (mapping segment length = 500 Kb, and perc_identity = 75). The genomic sequences of three Carangidae fish showed synteny to pompano genome (Fig. 7a–c). We found that the 24 pseudo-chromosomes of *Echeneis naucrates* had clear one-to-one relationship to pompano pseudo-chromosomes (Fig. 7d), suggesting that these two genomes did not experience chromosome fission and fusion events. These results revealed that the pompano genome will contribute to the study of the genome evolution of the Carangidae family and the Carangiformes order.

## Data Records

All sequencing data, genome assembly, predicted gene models and functional annotation were deposited in public repositories. The Illumina genomic sequencing reads, Pacbio long reads, Hi-C data, and RNA-seq reads of eight tissues were deposited in Sequence Read Archive at NCBI SRP136697^54^. The chromosome-level assembly was available in the GenBank at NCBI UWUD01000000^55^. The assembled contig, scaffolds, gene structure, homologs, and functional annotations were stored in Figshare^56^.

## Technical Validation

Three metrics, including peak length, total amount, and concentration were used to estimate the degradation level and quality of DNA samples. To construct Illumina libraries, the peak length of the isolated DNA was ≥20 kb and total DNA ≥5 μg with minimum 50 ng/μL. For PacBio libraries, the peak length was ≥40 kb and total DNA ≥7 μg with minimum 70 ng/μL. To construct the RNA-seq library of each tissue, the RNA integrity was ≥7.0 and total RNA ≥10 μg with rRNA ratio ≥1.5.

## Data Availability

Canu in the genome assembly and BLAT alignment in the gene prediction were utilized with specific parameters, described in Methods. The other bioinformatics tools were run with the default parameters. There were no any custom specific codes.
